# Effects of Glucagon-Like Peptide-1 Receptor Agonists on Bone Metabolism in Type 2 Diabetes Mellitus: A Systematic Review and Meta-Analysis

**DOI:** 10.1155/2024/1785321

**Published:** 2024-09-14

**Authors:** Xin Li, Yang Li, Chen Lei

**Affiliations:** ^1^ Department of Nutrition General Hospital of Ningxia Medical University, Yinchuan, 750004, Ningxia, China; ^2^ Department of Geriatrics and Special Needs General Hospital of Ningxia Medical University, Yinchuan, 750004, Ningxia, China

## Abstract

**Background:**

Glucagon-like peptide-1 receptor agonists (GLP-1 RAs) are an intriguing class of antihyperglycemic drugs for type 2 diabetes mellitus (T2DM). Such drugs not only play a primary role in regulating blood glucose levels but also exhibit additional pleiotropic effects, including potential impacts on bone metabolism and fracture risk. However, the mechanism of such drugs is unclear. The purpose of this study was to evaluate the effect of GLP-1 RAs on bone metabolism in T2DM.

**Methods:**

From database inception to May 1, 2023, the searches were conducted on multiple databases such as Web of Science, Embase, PubMed, CNKI, the Cochrane Library, Wanfang, and VIP. We systematically collected all randomized controlled trials of bone metabolism in patients with T2DM treated with GLP-1 RAs. The quality evaluation was performed according to the Cochrane Handbook for Systematic Reviews of Interventions. Data extraction was analyzed using Review Manager 5.4 software, and funnel plots were drawn to evaluate publication bias.

**Results:**

Twenty-six randomized controlled trials that met the inclusion criteria were included, involving a total of 2268 participants. In this study, compared to other antidiabetic drugs or placebo, GLP-1 RAs were found to significantly increase serum calcium (mean difference (MD) = 0.05, 95% confidence interval (CI) (0.01, 0.09), *P* = 0.002], bone alkaline phosphatase [standardized MD (SMD) = 0.76, 95% CI (0.29, 1.24), and *P* = 0.001), and osteocalcin (SMD = 2.04, 95% CI (0.99, 3.08), and *P* = 0.0001) in T2DM. Specifically, liraglutide increased procollagen type 1 N-terminal propeptide (SMD = 0.45, 95% CI (0.01, 0.89), and *P* = 0.04). GLP-1 RAs were also associated with a reduction in cross-linked C-terminal telopeptides of type I collagen (SMD = −0.36, 95% CI (−0.70, −0.03), and *P* = 0.03). In additionally, GLP-1 RAs increased lumbar spine bone mineral density (BMD) (SMD = 1.04, 95% CI (0.60, 1.48), and *P* < 0.00001) and femoral neck BMD (SMD = 1.29, 95% CI (0.36, 2.23), and *P* = 0.007).

**Conclusions:**

GLP-1 RAs can not only improve BMD in the lumbar spine and femoral neck of patients with T2DM but also protect bone health by inhibiting bone resorption and promoting bone formation. *Systematic Review Registration*. PROSPERO, identifier CRD42023418166.

## 1. Background

Type 2 diabetes mellitus (T2DM) is the most common endocrine disorder. Due to long-term exposure to high blood glucose, patients can develop a series of complications, mainly affecting the major blood vessels of the heart, microvessels in the kidneys and retina, as well as the nervous and skeletal systems [1]. Such conditions exert a substantial impact on the quality of life for patients and their families.

In recent times, T2DM is increasingly recognized as a significant contributor to secondary osteoporosis and fragility fractures in the skeletal system [2]. In clinical practice, several commonly prescribed antidiabetic medications not only contribute to controlling the blood glucose level of patients but also have diverse effects on their bone health. For instance, a study has indicated that metformin plays a beneficial role in promoting bone formation and improving bone metabolism [3]. However, as shown in a study, metformin does not have a substantial impact on enhancing bone mineral density (BMD) in patients with T2DM [4]. Conversely, thiazolidinedione drugs have been found to induce osteoblast apoptosis, leading to reduced bone formation and an increased risk of fractures [5].

Glucagon-like peptide-1 receptor agonists (GLP-1 RAs) are a novel class of antidiabetic medications favored by T2DM patients due to their dual benefits of lowering blood glucose levels and promoting weight loss [6]. According to a study, GLP-1 RAs can reduce bone breakdown by affecting osteoclasts, thereby inhibiting bone resorption. Besides, GLP-1 RAs can enhance osteoblast activity and promote bone formation [7]. Furthermore, GLP-1 RAs can control blood glucose levels positively, thus influencing bone health. Such processes exert an antiosteoporotic role [8]. Although GLP-1 RAs can inhibit bone resorption, stimulate bone formation, enhance BMD, and improve overall bone quality [8], the impact of GLP-1 RAs on fracture risk remains highly controversial. A meta-analysis demonstrated that compared to other antidiabetic medications, the use of GLP-1 RAs does not result in a reduction in fracture risk among patients with T2DM [9]. Nevertheless, a separate network meta-analysis published in 2018 indicated that GLP-1 RAs significantly decrease the risk of fractures in patients with T2DM compared with placebo or other antidiabetic medications [10].

As a result, the impact of GLP-1 RAs on fracture risk and their influence on sensitive bone metabolism markers remain uncertain. In addition, differences in the structure and duration of action among various GLP-1 RAs may contribute to variations in their effects. Currently, comprehensive meta-analyses focusing on the relationship between GLP-1 RAs and fracture risk are very limited, and the effects of these medications on bone metabolism markers and BMD have not been extensively studied in meta-analyses. Therefore, the purpose of this study was to systematically evaluate and analyze the effects of GLP-1 RAs on selected bone metabolism markers and BMD in T2DM.

## 2. Methods

### 2.1. Protocol and Registration

The protocol for this systematic review and meta-analysis has been registered with PROSPERO (registration number: CRD42023418166). Following the Preferred Reporting Items for Systematic Reviews and Meta-Analyses (PRISMA) statement [11], we present our methods and findings (Supplemental Table 1).

### 2.2. Eligibility Criteria

Randomized controlled trials (RCTs) were included to compare the efficacy of GLP-1 RAs with other antidiabetic medications or placebo. Each trial included participants who were aged 18 years or older and diagnosed with T2DM according to the diagnostic criteria of the World Health Organization or the American Diabetes Association if glycated hemoglobin (A1C) reaches or exceeds 6.5%, or if fasting plasma glucose levels reach or exceed 126 mg/dL (7.0 mmol/L), or if the 2-hour plasma glucose during an oral glucose tolerance test (OGTT) reaches or exceeds 200 mg/dL (11.1 mmol/L), or if random plasma glucose levels at any time reach or exceed 200 mg/dL (11.1 mmol/L) accompanied by typical symptoms of diabetes, the diagnosis of T2DM can be made based on any of these criteria [12]. There were no restrictions based on race, gender, or duration of the disease, and exclusion criteria did not consider the presence of osteoporosis or a history of fractures. The primary outcomes of interest were BMD or bone metabolism markers. In the case of multiple publications from the same study, we selected the data that provided the most comprehensive and longest follow-up period.

### 2.3. Search Strategy

We searched PubMed, Cochrane, Embase, Web of Science, CNKI, Wanfang, and VIP for relevant literature from the database inception to May 1, 2023. Detailed information regarding our search strategy is presented in the electronic supplementary material (Supplemental Table 2). To ensure inclusiveness, we included search terms related to “GLP-1 RAs” as well as specific terminologies of different types of GLP-1 RAs such as semaglutide, liraglutide, exenatide, dulaglutide, and benaglutide to capture all potentially eligible studies and avoid any omissions.

### 2.4. Selection Process

All search results were imported into EndNote (version X9, Thomson Reuters, Philadelphia, PA, USA) to remove any duplicate records. Two reviewers independently conducted an initial screening based on the title and abstract of each article. Subsequently, the remaining articles underwent a full-text assessment to determine their eligibility for inclusion, with reasons for exclusion carefully documented. In case of disagreements, a third reviewer was consulted to reach a consensus. Articles that did not provide the necessary data were excluded, as well as those for which the required data could not be obtained even after contacting the corresponding authors.

### 2.5. Data Collection and Risk of Bias Assessment

Two researchers independently extracted the relevant data. The included data consisted of the first author's name, publication year, country where the study was conducted, sample sizes and relevant information for the treatment and control groups, names of GLP-1 RAs, dosage, duration of the study, and results of bone metabolism-related markers before and after treatment. In cases of differences of opinion during the extraction process, the resolution was achieved through discussion or involvement of a third researcher. The Review Manager 5.4 software was used by the two researchers to assess the risk of bias in the included RCTs. This assessment was conducted using the bias risk assessment tool provided in the Cochrane Handbook for Systematic Reviews of Interventions, version 5.3 [13].

### 2.6. Statistical Analysis

The relevant outcome markers reflecting bone metabolism and BMD collected in this study were all continuous variables. Therefore, the mean difference (MD) or standardized mean difference (SMD) with standard deviation (SD) was chosen as effect markers. Heterogeneity between included studies was evaluated using the *Q* test and *I*^2^ statistic. The *Q* test primarily was used to assess the *p* value, and if the result of the heterogeneity test was *p* ≥ 0.1 and *I*^2^ < 50%, it indicated that there was no statistically significant heterogeneity among the studies, and a fixed-effect model was used for meta-analysis. Otherwise, the subgroup analysis could be performed to identify the source of heterogeneity or a random-effects model could be used to pool the effect sizes for meta-analysis. Sensitivity analysis was conducted to assess the stability of the results by sequentially excluding individual studies and reanalyzing the data. If the exclusion of a particular study led to significant changes in the pooled effect size or its heterogeneity, further reading and evaluation of that study were necessary. Publication bias was assessed by a visual funnel plot of the main outcome markers. Statistical analysis of all predetermined outcome markers was performed using RevMan 5.4 provided by the Cochrane Collaboration. *P* < 0.05 was considered statistically significant.

## 3. Results

### 3.1. Search Results

**Figure 1:**
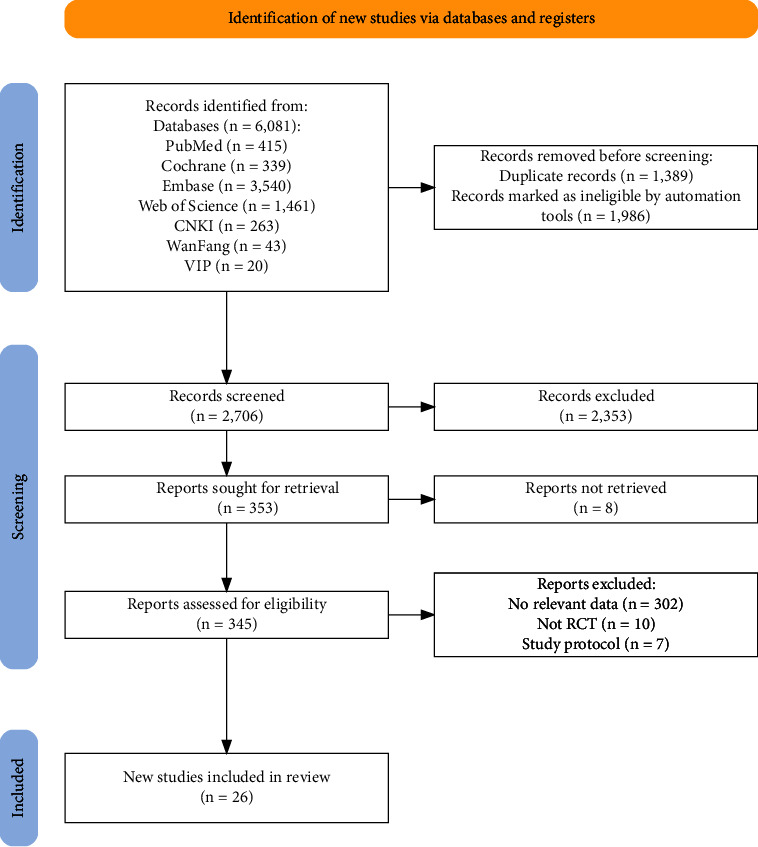
Literature screening process flowchart.

According to the established retrieval strategy, a total of 6081 studies were screened from 7 databases. Besides, EndNote X9 was used to remove 1389 duplicate records and 1986 records were automatically marked as ineligible. Then, we read the titles and abstracts of the remaining articles based on the inclusion and exclusion criteria, ultimately excluding 2353 articles. Among the remaining 353 articles, 8 were inaccessible in full text. After thoroughly reading the full texts, we found that 302 articles did not contain the required outcome markers, 10 were not RCTs, and 7 were study protocols. Therefore, 26 studies were ultimately included [14–38], involving a total of 2268 participants (Figure 1).

### 3.2. Study Characteristics

The analysis included a total of 26 RCTs conducted in English or Chinese. These studies encompassed a wide age range of patients. The patients in these studies ranged in age from 18 to 80 years old, with the treatment duration varying from 4 weeks to 104 weeks. The investigated GLP-1 RAs in these 26 studies consisted of liraglutide, exenatide, dulaglutide, and benaglutide. Specifically, 21 studies focused on liraglutide [14–33, 39], 3 on exenatide [34–36], 1 on the combination of exenatide and dulaglutide [37], and 1 study on benaglutide [38]. For a comprehensive understanding of the included studies, the detailed characteristics were shown in Table 1. Summary of findings is provided in Supplemental Table 3.

**Figure 2:**
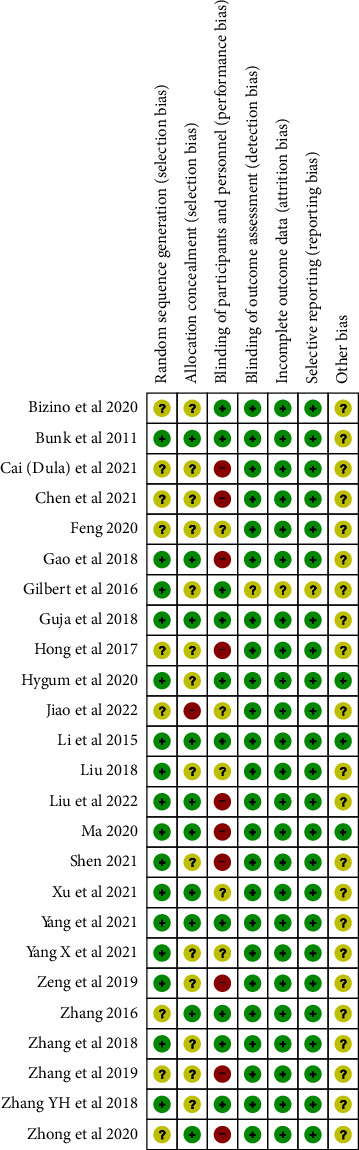
Risk of bias summary.

Of the 25 studies included, 16 studies provided detailed descriptions of the methods used for random sequence generation, and the remaining 9 studies mentioned randomization without specifying the exact methods used. There were 10 studies explicitly stating the allocation concealment method, and 10 studies in blinding for participants and personnel, as well as 24 studies on outcome assessors. The risk of bias assessment for each study was presented in Figure 2.

### 3.3. Bone Mineral Density

**Figure 3:**
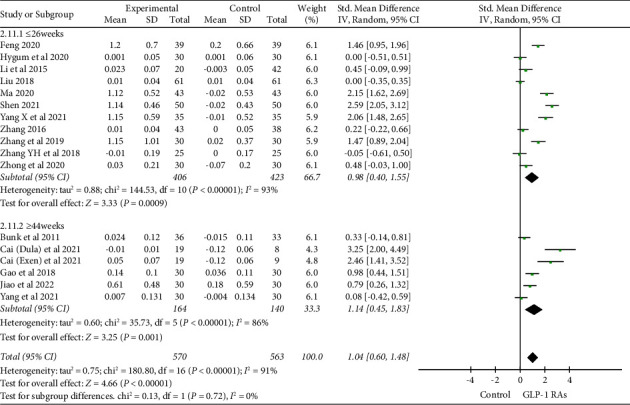
Comparison of lumbar spine BMD in the GLP-1 RAs group compared with the control group.

A total of 16 studies [16, 17, 19, 21–23, 25–29, 32, 34, 36–38] have examined the effects of GLP-1 RAs on lumbar spine BMD in patients with T2DM. The meta-analysis demonstrated a significant increase in lumbar spine BMD in the GLP-1 RAs treatment group compared to the control group (SMD = 1.04, 95% confidence interval (CI) (0.60, 1.48) and *P* < 0.00001). A subgroup analysis based on treatment duration revealed that, in comparison with the control group, treatment durations of ≤26 weeks exhibited a combined effect size for lumbar spine BMD (SMD = 0.98 and 95% CI (0.40, 1.55)). Besides, treatment durations of ≥44 weeks showed a combined effect size for lumbar spine BMD (SMD = 1.14 and 95% CI (0.45, 1.83)) (Figure 3). These findings suggested that both shorter (≤26 weeks) and longer (≥44 weeks) treatment durations could enhance lumbar spine BMD, with a more pronounced improvement observed with longer treatment duration.

**Figure 4:**
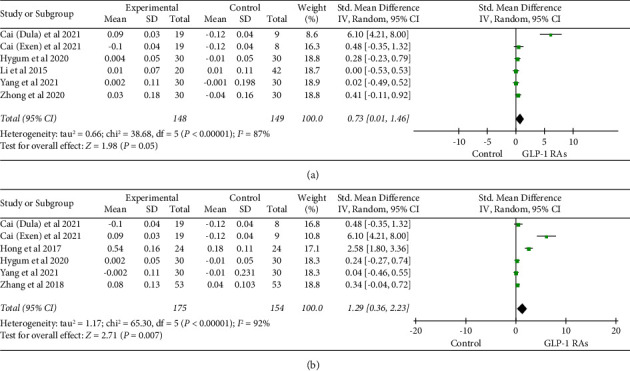
Comparison of total hip BMD (a) and femoral neck BMD (b) in the GLP-1 RAs group compared with the control group.

We conducted five studies [19, 25, 29, 36, 37] to investigate the impact of GLP-1 RAs on total hip BMD in T2DM. In contrast to the control group, total hip BMD was slightly increased in the GLP-1 RAs treatment group (SMD = 0.73, 95% CI (0.01, 1.46), and *P*=0.05). However, a statistically significant difference was not observed between the two groups (Figure 4(a)).

Five studies [19, 20, 25, 33, 37] reported the effects of GLP-1 RAs on femoral neck BMD in patients with T2DM. The results revealed that the GLP-1 Ras group exhibited a significant increase in femoral neck BMD as opposed to the control group (SMD = 1.29, 95% CI (0.36, 2.23), and *P*=0.007) (Figure 4(b)).

### 3.4. Bone Metabolism

**Figure 5:**
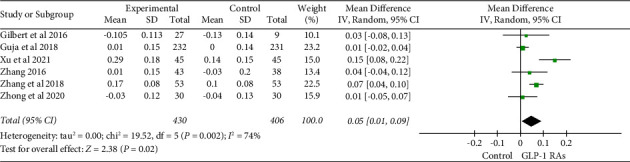
Comparison of serum calcium in the GLP-1 RAs group compared with the control group.

Six studies [18, 24, 28, 29, 33, 35] exhibited the effects of GLP-1 RAs on serum calcium in patients with T2DM. The results showed that compared with the control group, GLP-1 RAs improved the level of serum calcium (MD = 0.05, 95% CI (0.01, 0.09), and *P*=0.002) (Figure 5).

**Figure 6:**
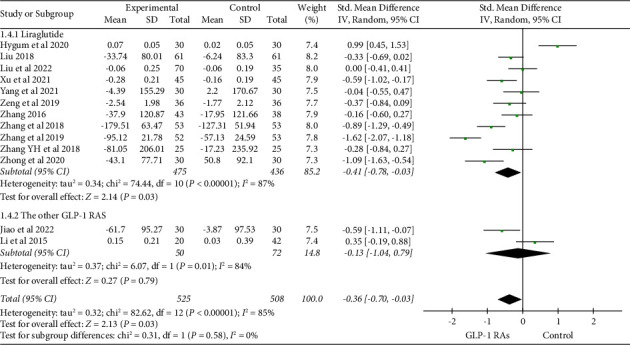
Comparison of CTX in the GLP-1 RAs group compared with the control group.

The combined analysis of 13 studies [19, 21, 24, 25, 27–30, 32, 33, 36, 38] suggested that GLP-1 RAs are more effective in reducing cross-linked C-terminal telopeptides of type I collagen (CTX) in comparison with the control group (SMD = −0.36, 95% CI (−0.70, −0.03), and *P*=0.03). A subgroup analysis based on medication type displayed that as opposed to other GLP-1 RAs, liraglutide has a statistically significant effect in reducing CTX levels (Figure 6).

**Figure 7:**
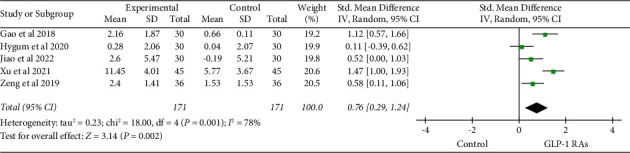
Comparison of BALP in the GLP-1 RAs group compared with the control group.

**Figure 8:**
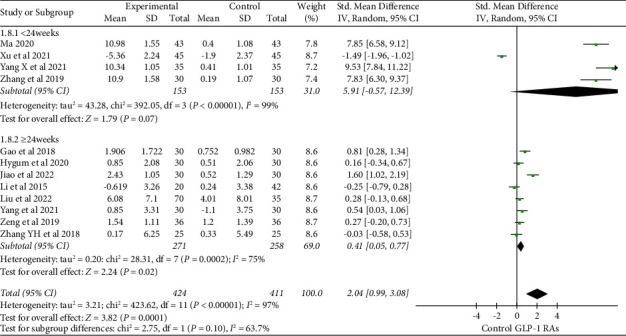
Effect of GLP-1 RAs on osteocalcin under different treatment durations.

**Figure 9:**
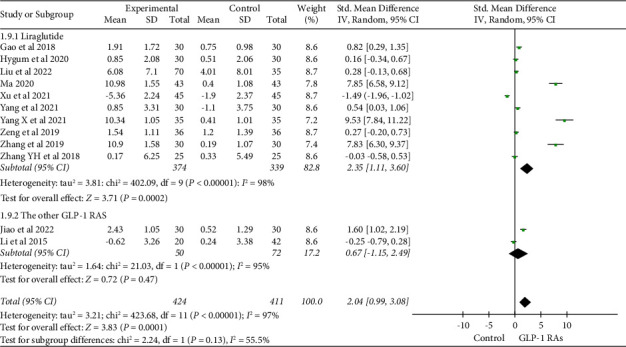
Effects of different types of GLP-1 RAs on osteocalcin.

The effect of GLP-1 RAs on BALP was found in 5 studies [17, 19, 24, 30, 38]. In contrast to the control group, there was a significant increase in the effect of GLP-1 RAs on bone alkaline phosphatase (BALP) (SMD = 0.76, 95% CI (0.29, 1.24), and *P*=0.002) (Figure 7). After analyzing 12 studies [17, 19, 22, 24–26, 30–32, 36, 38] including osteocalcin, it was found that GLP-1 RAs significantly improved the level of osteocalcin compared to the control group (SMD = 2.04, 95% CI (0.99, 3.08), and *P*=0.0001). The subgroup analysis based on treatment duration demonstrated the combined effect size of osteocalcin (SMD = 0.41, 95% CI (0.05, 0.77), and *P*=0.02) when the treatment duration was ≥24 weeks. Such a result indicated a statistically significant difference (Figure 8). According to a subgroup analysis based on the type of medication, liraglutide markedly improved the osteocalcin level (SMD = 2.35 and 95% CI (1.11, 3.60)) (Figure 9).

**Figure 10:**
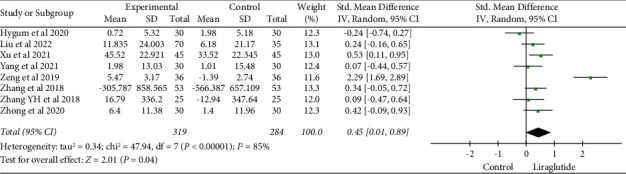
Comparison of P1NP in the liraglutide group compared with the control group.

Eight studies [19, 24, 25, 29, 30, 32, 33] have represented the effects of GLP-1 RAs on procollagen type 1 N-terminal propeptide (P1NP). The abovementioned studies suggested that liraglutide can be used as a treatment method. The results showed that compared with the control group, liraglutide exhibited an improvement in P1NP (SMD = 0.45, 95% CI (0.01, 0.89), and *P*=0.04). The difference between the two groups was statistically significant (Figure 10).

No significant differences were observed between the GLP-1 RAs group and the control group in serum phosphate, 25-hydroxyvitamin D, and tartrate resistant acid phosphatase-5b (TRACP-5b) (Supplemental Figures 1–3).

In addition, a sensitivity analysis was performed by systematically excluding each study, and no significant change was found in the overall effect size and heterogeneity. Funnel plots were created to assess the impact of GLP-1 RAs on CTX and lumbar spine BMD (Supplemental Figures 4 and 5). The plots displayed a symmetrical distribution of studies on both sides of the axis, indicating a relatively low risk of publication bias.

## 4. Discussion

Ultimately, GLP-1 RAs played a beneficial effect on BMD and bone metabolism in patients with T2DM. Compared to other antidiabetic drugs or placebo, GLP-1 RAs may demonstrate greater potential benefits for bone health in the treatment of T2DM. Specifically, dipeptidyl peptidase-4 (DPP-4) inhibitors and GLP-1 RAs share similar mechanisms of action. However, some studies suggest that DPP-4 inhibitors have a neutral or mildly positive effect on bone health [40]. In contrast, clinical trials have demonstrated more pronounced effects of GLP-1 RAs in reducing glycated hemoglobin and body weight, making them a more favorable option for most patients [41]. Furthermore, insulin therapy for diabetes may alter levels of certain biomarkers associated with bone metabolism, such as advanced glycation end products and other indicators related to bone density and fracture risk, thereby influencing the processes of bone formation and resorption [42]. Our study also concluded that the longer treatment duration was associated with the more significant improvements in lumbar spine BMD. Furthermore, CTX could be reduced and BALP, osteocalcin, as well as P1NP, could be increased. These findings indicated that GLP-1 RAs could suppress bone resorption and promote bone formation in T2DM. However, notably, the studies included in this analysis primarily focused on liraglutide and no statistically significant changes were revealed in subgroup analyses for other GLP-1 RAs. This could be attributed to the limited number of studies and sample sizes for other GLP-1 RAs, as well as potential differences in molecular structure between exenatide and dulaglutide in contrast to liraglutide.

In recent years, GLP-1 RAs have attracted much attention as an antidiabetic medication. As shown in the study, these drugs not only reduced glucose sugar levels and had a cardiovascular protective effect but also may have a certain protective effect on bone health. According to the animal experiments, as opposed to the wild-type control group, GLP-1 RAs knockout mice exhibit an increase in osteoclast numbers as well as bone resorption levels, and a decrease in BMD [43]. In a mouse model induced by lipopolysaccharide, the combined treatment with GLP-1 RAs significantly reduces osteoclast numbers and CTX in comparison with mice receiving lipopolysaccharide alone [44]. As reported by Sedky, GLP-1 RAs can enhance osteocalcin in diabetic rats, thereby increasing bone mass and strength [45]. These animal studies indicated that GLP-1 RAs can inhibit bone resorption, promote bone formation, and improve BMD. Such results were consistent with the findings of this study.

However, there was still no definitive and consistent conclusion from existing clinical studies on the impact of GLP-1 RAs on bone metabolism markers and fracture risk in patients with T2DM. In a clinical trial published by Gilbert in 2016 [18], the trial lasting for 104 weeks demonstrated that liraglutide as monotherapy does not affect total BMD in patients. However, the dropout rate of this trial is as high as 52%, which may greatly affect the final results and may be the reason for the inconsistency with our study findings. Zhang [28] pointed out that liraglutide had no significant effect on whole-body BMD and bone formation markers in obese and overweight patients with T2DM after 26 weeks of treatment. However, it does reduce the level of the bone resorption marker CTX, which was consistent with the results of our study. The difference in results might be only obese and overweight patients with T2DM were included in the clinical study, and the low-grade inflammatory state in patients with obesity also affected bone metabolism [46, 47]. In addition, some studies have suggested that exenatide may increase fracture risk. However, this meta-analysis only assessed fractures as adverse events without including detailed bone metabolism markers or bone quality indicators, such as bone resorption and formation markers, BMD, and calcium and phosphate levels [48]. In contrast, our study provided a detailed analysis of these markers, enabling a more accurate evaluation of the effects of GLP-1 RAs on bone health. Overall, although the analyses suggest that liraglutide may reduce fracture risk in patients with T2DM, patients using GLP-1 RAs do not exhibit a significant increase in fracture risk; in some cases, GLP-1 RAs may be associated with a lower fracture risk. Compared to other antidiabetic medications, GLP-1 RAs generally show a safer profile regarding fracture risk [49].

This meta-analysis has several limitations. First, most of the included RCTs primarily used liraglutide, while the number of RCTs for other GLP-1 RAs was limited, with smaller sample sizes, potentially affecting the comprehensiveness of the results. Second, the effects of the medication may take a longer time to manifest, with treatment durations in the studies ranging from 4 weeks to 104 weeks. Shorter treatment periods could impact the strength of the evidence. Third, we only included published clinical trials and did not include unpublished trials, which may introduce publication bias and omit relevant data. Furthermore, although the heterogeneity of results was high, sensitivity analyses were performed by systematically excluding each study to assess their impact on heterogeneity and overall effect size, with findings indicating that these exclusions did not significantly alter the overall effect size or heterogeneity. Finally, it is noteworthy that despite using multiple international databases to ensure comprehensive data retrieval, regional bias may still be present. For instance, studies from Western countries generally suggest that GLP-1 receptor agonists have no significant impact on BMD or bone turnover markers, while studies from China have shown potential positive effects on bone health. This discrepancy may be related to differences in dietary habits, genetic variations, healthcare resources, and research methodologies. Future research should consider including studies from diverse regions or performing sensitivity analyses to exclude Chinese publications to more comprehensively assess the impact of GLP-1 RAs on bone metabolism.

In summary, in this study, GLP-1 RAs can effectively suppress bone resorption by reducing CTX in patients with T2DM compared to other antidiabetic medications or placebo. Besides, GLP-1 RAs also promote bone formation by increasing BALP, P1NP, and osteocalcin. In addition, GLP-1 RAs can not only improve BMD at the lumbar spine and femoral neck but also enhance bone quality. Such results can delay the occurrence and progression of osteoporosis and reduce the risk of fractures in patients with T2DM. Among the GLP-1 RAs, liraglutide seems to have more effective effects in reducing CTX and increasing osteocalcin as opposed to exenatide and dulaglutide. Patients with T2DM are already at high risk of osteoporosis or fractures, so it is important to choose antidiabetic medications that not only lower blood glucose but also minimize the risk of osteoporosis or fractures.

## Figures and Tables

**Table 1:** Characteristics of included studies.

Study	GLP-1 Ras	Control	Sample size	Male (%)	Mean age (year)	Mean HbA1c (%)	Mean BMI (kg/m^2^)	Follow-up (weeks)
Bizino et al., 2020 [14]	Lira 1.8 mg QD	Placebo	49	59.2	59.5	8.3	32.1	26
Chen et al., 2021 [15]	Lira 1.8 mg QD	Dapagliflozin	40	77.5	45.5	9.4	NR	12
Feng, 2020 [16]	Lira 1.2 mg QD	Metformin	78	57.7	69.8	10.3	NR	4
Gao et al., 2018 [17]	Lira 1.2 mg QD	Metformin	60	100.0	60.8	8.9	28.7	48
Gilbert et al., 2016 [18]	Lira 1.2 mg/1.8 mg QD	Glimepiride	61	45.9	54.3	8.1	33.0	104
Hygum et al., 2020 [19]	Lira 1.8 mg QD	Placebo	60	50	63.0	6.6	32.2	26
Hong et al., 2017 [20]	Lira 1.8 mg QD	Metformin	48	NR	18–60	NR	NR	26
Liu, 2018 [21]	Lira 1.2 mg QD	Metformin	122	57.4	47.7	8.8	29.1	12
Liu et al., 2022 [39]	Lira 1.2 mg/1.8 mg QD	Metformin	105	52.4	61.3	8.1	25.3	36
Ma, 2020 [22]	Lira 1.2 mg QD	Metformin	86	50.0	67.4	10.2	24.6	4
Shen, 2021 [23]	Lira 1.2 mg QD	Metformin	100	55.0	69.9	10.4	24.2	8
Xu et al., 2021 [24]	Lira 1.8 mg QD	Insulin	90	53.3	67.3	7.6	N	12
Yang et al., 2021 [25]	Lira 1.2 mg QD	Insulin glargine	60	100.0	40.1	8.8	24.4	48
Yang et al., 2021 [26]	Lira 1.2 mg QD	Metformin	70	47.1	75.0	10.2	24.8	4
Zhang et al., 2019 [27]	Lira 1.2 mg QD	Metformin	60	N	66.2	10.4	25.0	4
Zhang, 2016 [28]	Lira 1.2 mg QD	Insulin Aspart30	81	35.8	51.2	8.9	29.5	26
Zhong et al., 2020 [29]	Lira 1.2 mg QD	Dapagliflozin	60	0.0	58.9	8.8	27.4	26
Zeng et al., 2019 [30]	Lira 1.2 mg QD	Metformin	72	100.0	48.0	8.9	27.1	48
Zhang et al., 2019 [31]	Lira 1.8 mg QD	Linagliptin	104	53.8	54.0	23.4	35.5	12
Zhang et al., 2018 [32]	Lira 1.2 mg QD	Metformin	50	46.0	44.0	8.4	26.0	24
Zhang et al., 2018 [33]	Lira 0.6 mg QD	Insulin Aspart30	106	56.6	65.3	7.6	28.6	12
Bunck et al., 2011 [34]	Exen 10 µg BID	Insulin glargine	69	65.2	58.4	7.5	30.5	44
Guja et al., 2018 [35]	Exen 2 mg QW	Placebo	461	47.9	57.7	8.5	33.7	28
Li et al., 2015 [36]	Exen 10 *µ*g BID	Insulin/pioglitazone	62	51.6	50.0	8.6	26.1	24
Cai et al., 2021 [37]	Exen 2 mg QW/Dula 1.5 mg QW	Insulin glargine/placebo	65	55.4	61.3	8.3	26.4	52
Jiao et al., 2022 [38]	Bena 0.1 mg TID	Insulin Aspart30	60	55.0	50.9	7.9	26.1	48

GLP-1 Ras, glucagon-like peptide-1 receptor agonists; BMI, bone mineral density; HbA1c, hemoglobin A1c; Lira, liraglutide; Exen, exenatide; Bena, benalutide; NR, not reported; QD, quaque die; BID, bis in die; QW, quaque week; TID, ter in die.

## Data Availability

The dataset used to support the findings of this study is available from the corresponding author upon reasonable request.

## Abbreviations

ALP: Alkaline phosphatase
BALP: Bone alkaline phosphatase
BMD: Bone mineral density
CTX: Cross-linked C-terminal telopeptides of type I collagen
DPP-4: Dipeptidyl peptidase-4
GLP-1 RAs: Glucagon-like peptide-1 receptor agonists
P1NP: Procollagen type 1 N-terminal propeptide
RCTs: Randomized controlled trials
SMD: Standard mean difference
T2DM: Type 2 diabetes mellitus
TRACP-5b: Tartrate resistant acid phosphatase-5b.

## Supplementary Materials

Supplementary Materials Supplemental Figure 1: comparison of serum phosphate in the GLP-1 RAs group compared with the control group. Supplemental Figure 2: comparison of 25-hydroxyvitamin D in the GLP-1 RAs group compared with the control group. Supplemental Figure 3: comparison of TRACP-5b in the GLP-1 RAs group compared with the control group. Supplemental Figure 4: funnel plot of the impact of GLP-1 RAs on lumbar spine bone mineral density. Supplemental Figure 5: funnel plot of the impact of GLP-1 RAs on CTX. Supplemental Table 1: PRISMA checklist. Supplemental Table 2: search Strategy. Supplemental Table 3: summary of Findings.
