# Supplementary material for: Effects of Glucagon-Like Peptide-1 Receptor Agonists on Bone Metabolism in Type 2 Diabetes Mellitus: A Systematic Review and Meta-Analysis
Source: Int J Endocrinol. 2024 Sep 14;2024:1785321. doi: 10.1155/2024/1785321 (PMC11416174; doi:10.1155/2024/1785321)

**Supplemental Figure 1.** Comparison of serum phosphate in the GLP-1 RAs group compared with the control group


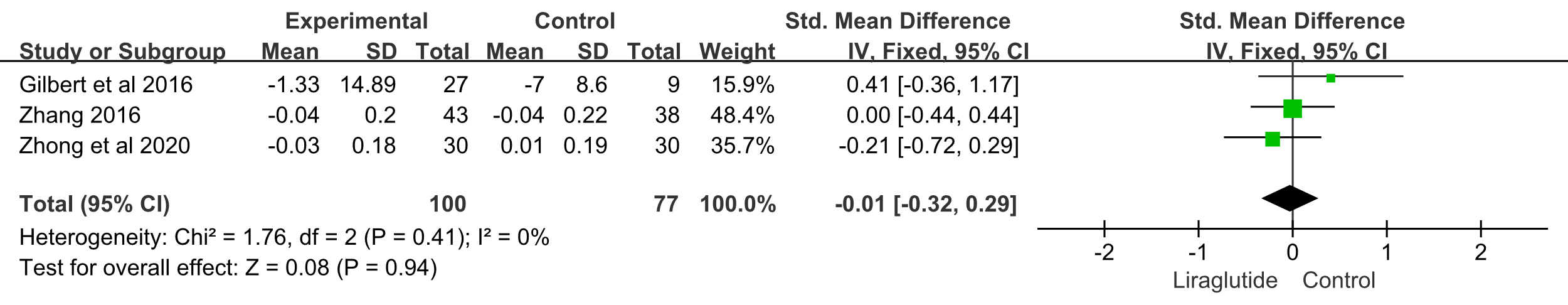


**Supplemental Figure 2.** Comparison of 25-hydroxyvitamin D in the GLP-1 RAs group compared with the control group
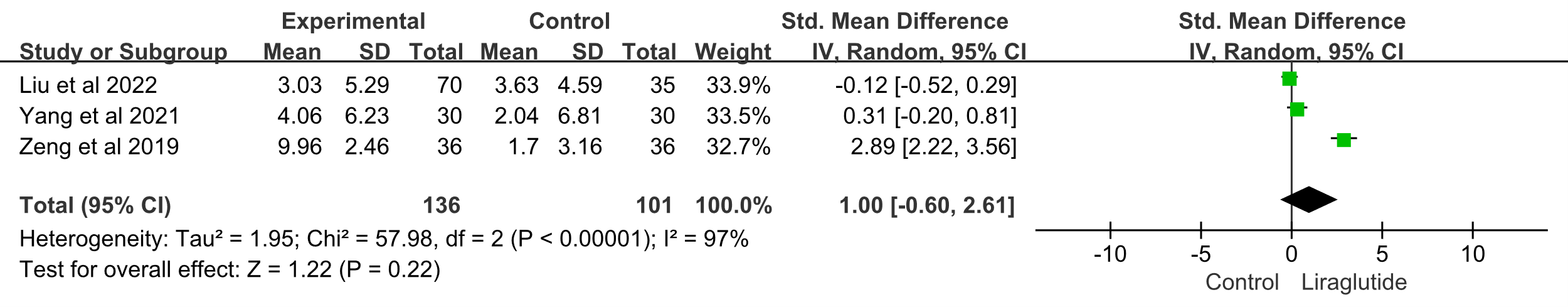


**Supplemental Figure 3.** Comparison of TRACP-5b in the GLP-1 RAs group compared with the control group


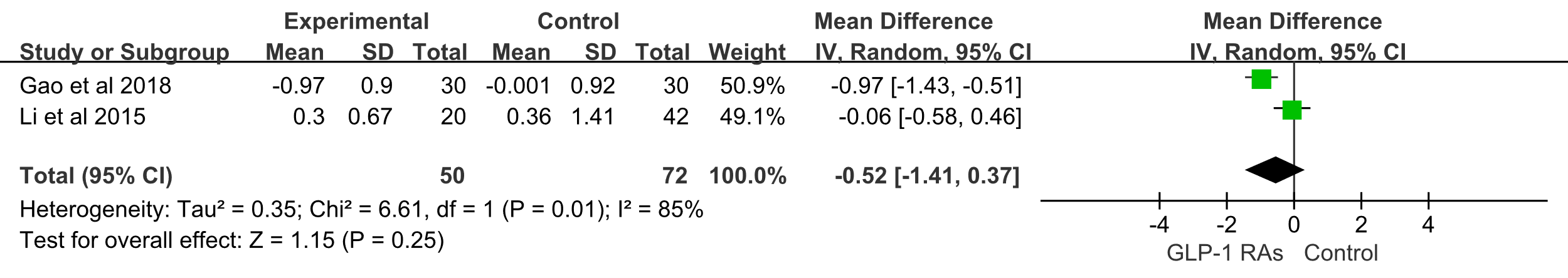


**Supplemental Figure 4.** Funnel plot of the impact of GLP-1 RAs on lumbar spine BMD.


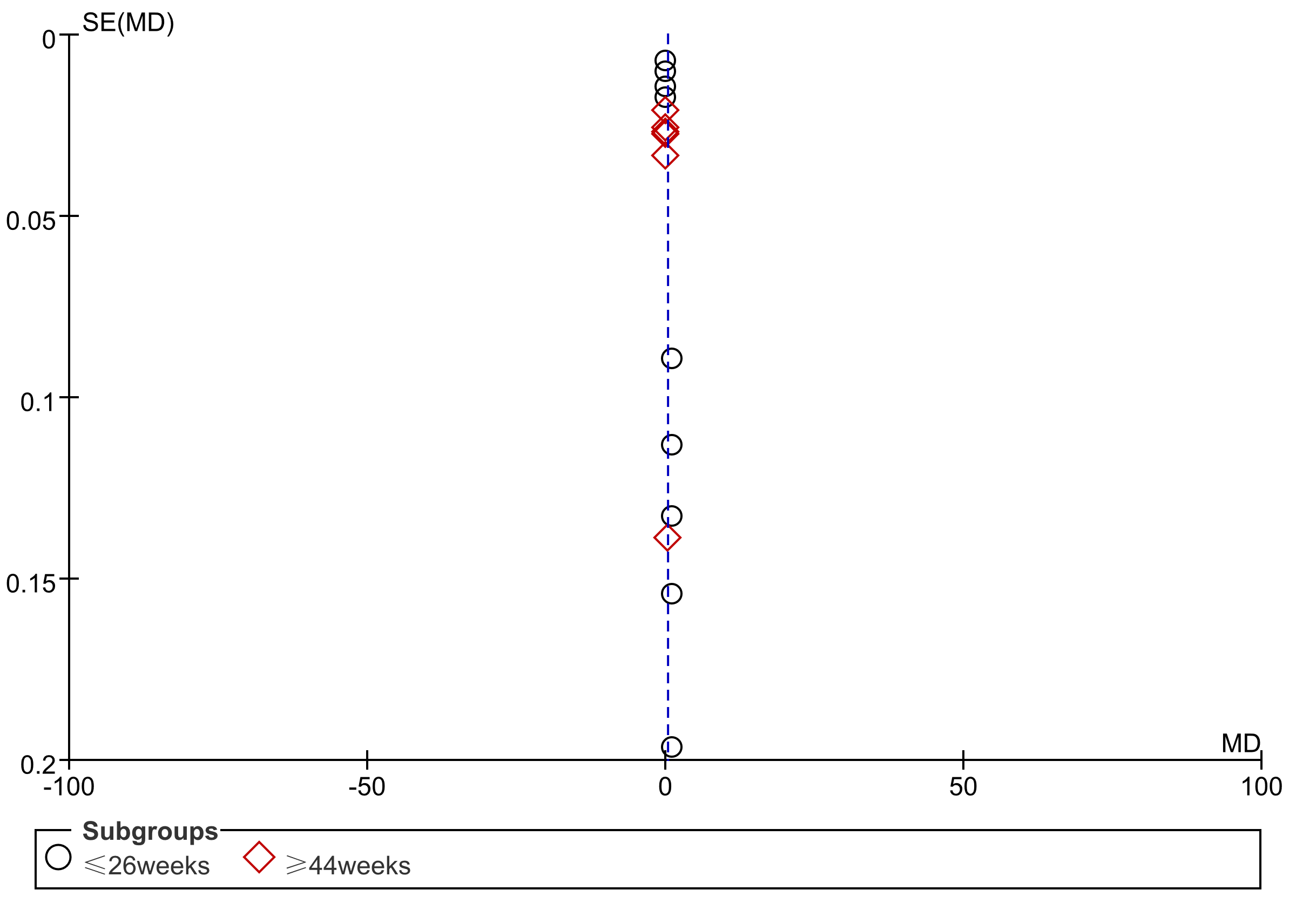


**Supplemental Figure 5.** Funnel plot of the impact of GLP-1 RAs on CTX.


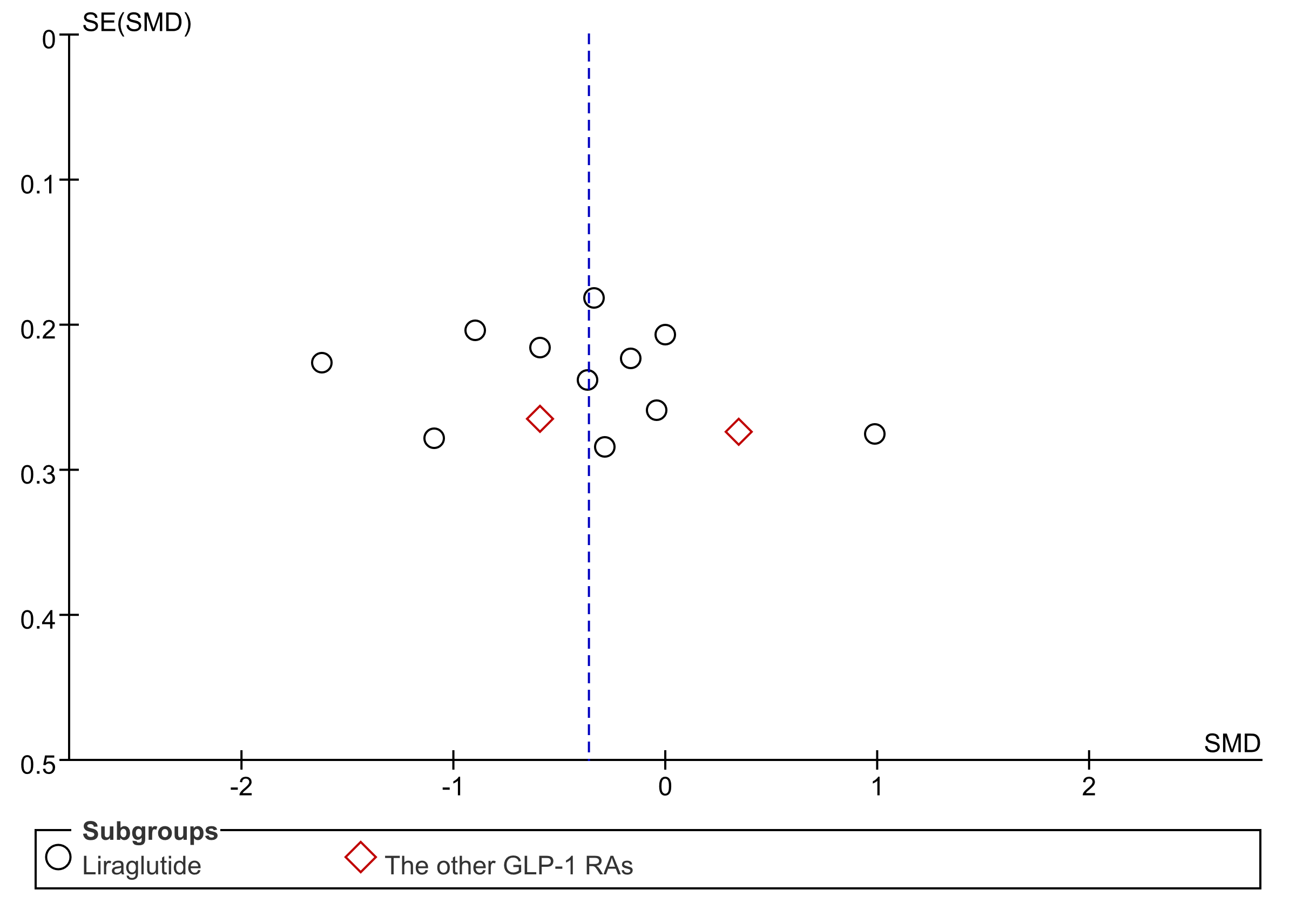

Supplement: Supplementary Materials — Supplemental Figure 1: comparison of serum phosphate in the GLP-1 RAs group compared with the control group. Supplemental Figure 2: comparison of 25-hydroxyvitamin D in the GLP-1 RAs group compared with the control group. Supplemental Figure 3: comparison of TRACP-5b in the GLP-1 RAs group compared with the control group. Supplemental Figure 4: funnel plot of the impact of GLP-1 RAs on lumbar spine bone mineral density. Supplemental Figure 5: funnel plot of the impact of GLP-1 RAs on CTX. Supplemental Table 1: PRISMA checklist. Supplemental Table 2: search Strategy. Supplemental Table 3: summary of Findings. [file 1785321.f1.zip › Supplemental Figures.docx]
